# Subjective Somatosensory Experiences Disclosed by Focused Attention: Cortical-Hippocampal-Insular and Amygdala Contributions

**DOI:** 10.1371/journal.pone.0104721

**Published:** 2014-08-28

**Authors:** Clemens C. C. Bauer, Fernando A. Barrios, José-Luis Díaz

**Affiliations:** 1 Laboratorio de Neuroimagen Functional, Instituto de Neurobiología, Universidad Nacional Autónoma de México, Querétaro, México; 2 Departamento de Historia y Filosofía de la Medicina, Facultad de Medicina, Universidad Nacional Autónoma de México, México, D. F., México; Emory University, United States of America

## Abstract

In order to explore the neurobiological foundations of qualitative subjective experiences, the present study was designed to correlate objective third-person brain fMRI measures with subjective first-person identification and scaling of local, subtle, and specific somatosensory sensations, obtained directly after the imaging procedure. Thus, thirty-four volunteers were instructed to focus and sustain their attention to either provoked or spontaneous sensations of each thumb during the fMRI procedure. By means of a Likert scale applied immediately afterwards, the participants recalled and evaluated the intensity of their attention and identified specific somatosensory sensations (e.g. *pulsation*, *vibration*, *heat*). Using the subject's subjective scores as covariates to model both attention intensity and general somatosensory experiences regressors, the whole-brain random effect analyses revealed activations in the frontopolar prefrontal cortex (BA10), primary somatosensory cortex (BA1), premotor cortex (BA 6), precuneus (BA 7), temporopolar cortex (BA 38), inferior parietal lobe (BA 39), hippocampus, insula and amygdala. Furthermore, BA10 showed differential activity, with ventral BA10 correlating exclusively with attention (r(32) = 0.54, p = 0.0013) and dorsal BA10 correlating exclusively with somatosensory sensation (r(32) = 0.46, p = 0.007). All other reported brain areas showed significant positive correlations solely with subjective somatosensory experiences reports. These results provide evidence that the frontopolar prefrontal cortex has dissociable functions depending on specific cognitive demands; i.e. the dorsal portion of the frontopolar prefrontal cortex in conjunction with primary somatosensory cortex, temporopolar cortex, inferior parietal lobe, hippocampus, insula and amygdala are involved in the processing of spontaneous general subjective somatosensory experiences disclosed by focused and sustained attention.

## Introduction

Before attempting to explain how and why neurophysiological processes relate to consciousness traits, it seems necessary to find consistent correlations between subjective phenomenological features and brain activity patterns [1]. For example, it is now possible to correlate introspective evaluations of sensory aspects of subjective experience with imaged local brain activations [2]. Such *neurophenomenological* program depends on the development of dynamic approaches to cerebral activity in conjunction to standardized and rigorous measurements of subjective experience obtained from first-person reports [3], [4]. A particular difficulty concerning the subjective character of conscious experience is the neural substrate of sensorial *qualia* features such as color, sound, scent, taste, touch, pain, and the like [5]. It has been suggested that the ventral prefrontal cortex is necessary, but not sufficient, for the generation of subjective experiences [6]–[8] and that there may be different areas involved depending on their specific character (e.g. auditory, tactile, emotional) [9], [10]. Other studies also report signal increases in frontopolar prefrontal cortex during different self-referential processing tasks [11]–[13] and the magnitude and time course of its activation predicts whether information is consciously perceived or slips away unnoticed [6].

Bilateral activations of temporopolar cortex were found during object encoding, tactile perception and self-related processing [14]–[16]. Furthermore, the phenomenal character of perceiving some objects as different from others is associated with right temporopolar activation [15].

The ability to voluntarily direct, concentrate, and sustain attention can bring into focus and enhance bottom-up qualitative processes of either a somatosenory/external or proprioceptive/internal nature [17]–[21]. A form of insight meditation requiring sustained awareness of subtle somatic sensations spontaneously arising from different body parts increases parieto-occipital gamma activity, a marker for enhanced sensory awareness [22]. Tactile attention also biases the processing of selected stimuli relevant features by amplifying somatosensory cortex responses [23]. Attention towards particular somatic stimuli, in turn, selectively enhances domain-specific cortical representations that probably are determinant for their conscious perception [21], [24].

Based on previous studies implicating several brain regions in the generation of subjective experiences [6]–[8], [14], [15] and the evidence that top-down attention control can be used to define particular sensory targets [21], [22], [24], we hypothesized that focusing attention on subtle pre-reflective somatosensory experiences would activate frontopolar prefrontal and temporopolar cortices, and, specifically, that the objectively measured brain activity within these regions would correlate with subjective sensory experience reports.

## Materials and Methods

### Subjects

All subjects gave written informed consent for the experimental procedure, and the protocol follows the principles expressed in the Declaration of Helsinki and was authorized by The Bioethics Committee of the Neurobiology Institute (Comité de Bioética del Instituto de Neurobiología, Universidad Nacional Autónoma de México). After standard exclusion criteria for functional magnetic resonance imaging (fMRI) were applied, 37 healthy volunteers participated in the study (16 female and 21 male, mean age 35.58 years, SD 7.97, 14 left handed and 23 right handed). Subjects were evaluated with digital versions of the Symptom Checklist 90 and Edinburgh Inventory to exclude psychological and/or psychopathological symptoms, and to evaluate handedness [25], [26]. All subjects gave informed consent for the experimental procedure, and the protocol had IRB approval.

### Experimental design

**Figure 1:**
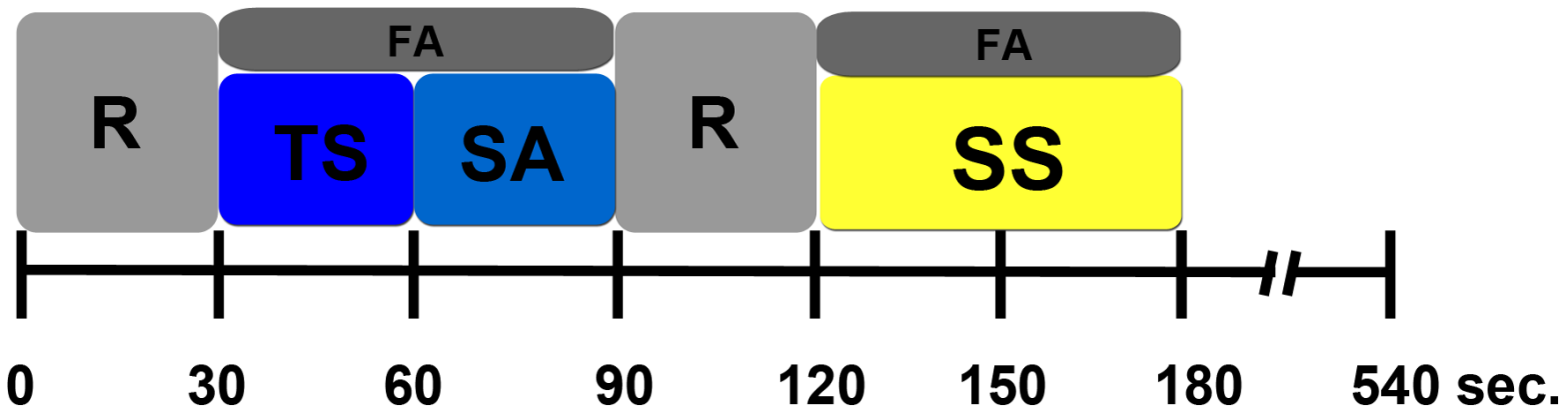
Single run experimental paradigm for either thumb. *Touch-Stimulus* (TS, in dark-blue), Stimulation-Aftereffect (SA, in light-blue) and *Spontaneous-Sensation* (SS, in yellow). Focusing attention (FA, in grey) was required during every condition. No attention task was required during resting periods between conditions (gaps).

Brain activation was examined during covert focused attention directed towards either the right or left thumb under two experimental conditions: (a) *External-Stimulus Condition* (manual caressing of either thumb with a 2-cm sponge brush at 1–2 Hz and stimulation aftereffect) and (b) S*pontaneous-Sensation Condition* in absence of any external stimulation (Figure 1). Resting periods without attention tasks separated both experimental conditions. Subjects were instructed to focus their attention on either thumb during the two experimental conditions and to abstain from moving it during the whole experiment. The instructions emphasized that, in the absence of touch stimuli, the subjects should focus their attention on the spontaneous sensations arising from either thumb rather than visualizing or imagining this body part. The protocol consisted of a block design paradigm alternating between focusing of attention towards the *External-Stimulus* of either thumb (60 sec blue block in Figure 1) or focusing of attention towards *Spontaneous-Sensation* of the same body part in the absence of external stimuli (60 sec yellow block in Figure 1). The length of the blocks was decided after a pilot study where the response showed that the subjects started to feel clear and distinct sensations ∼20–40 sec after the instruction. Right and left thumbs were run in separate procedures and the order of the thumb was randomly counterbalanced (Left thumb first 52%). The *External-Stimulus* block was further divided into a 30 sec *Touch-Stimulus Condition* (shown as a dark-block in Figure 1) and a 30 sec *Stimulation Aftereffect Condition* (shown as a light-blue block in Figure 1). *External-Stimulus* and *Spontaneous-Sensation* conditions were separated by 30 sec resting intervals to ensure no overlapping brain activity. Each run lasted 540 sec and consisted of three epochs. One epoch was a 180 sec sequence of Rest, Touch-Stimulus, Stimulation Aftereffect, Resting, and *Spontaneous-Sensation*. While in the scanner, the subjects received a previously agreed one-word instruction (“attention” or “rest”) via MRI compatible audio equipment (NordicNeuroLab, Bergen, Norway) directing them to focus their attention on the target thumb, or to rest. Subjects had their eyes closed during the whole experiment.

Immediately after the scanning procedure all subjects were submitted to a *Phenomenology Questionnaire* to assess first-person *Subjective Sensations* experienced during the *Spontaneous-Sensation Condition.* The *Phenomenology Questionnaire* was designed to reflect the participant's subjective assessment of their experience through all the blocks. It consisted of a *qualitative* free description of the experienced sensations followed by a *quantitative* section where attention strength and intensity of specific sensory *qualia* experienced across all *Spontaneous-Sensation* blocks were assessed by means of a 1 to 5 Likert scale (see below and Table 1 in Results section for details).

**Table 1:** Phenomenology Questionnaire.

	Qualitative Part		Quantitative Part										
Subject ID	*Freely Narrated exposure of subjective sensations*	First Thumb	*Attentional Strength & Specific Sensory Qualia experienced*										
		L	A	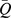	P	V	E	H	C	Sh	I	St	N
1	Pulsation, enlargement and tickling	L	5	3.67	5	5	5	1	1	1	5	5	5
2	I just felt it	R	3	2.00	1	1	3	4	3	2	1	1	2
3	Trembling, cold, heaviness	L	2	2.22	3	4	4	3	2	1	1	1	1
4	Light tickling	L	5	1.78	5	2	1	1	1	1	1	1	3
5	Tickling	RL	3	2.78	5	1	4	2	4	4	1	3	1
6	Heat and pulsation	L	2.5	2.22	4	3	3	3	1	2	1	1	2
7	A bit like a scratchy feeling, like a very porous sponge touching you, tickle	R	3.5	2.22	3	2	2	1	3	1	2	2	4
8	Increased awareness of the area regarding the sensory plates and dermatomas	R	5	1.89	1	1	1	2	1	1	4	4	2
9	I think I felt the air in the resonance room	R	3	2.22	3	2	3	4	1	1	1	1	4
10	Prickling	L	4.5	3.22	4	5	3	3	2	2	4	2	4
11	I felt something like palpitations	L	3	2.44	4	3	5	5	1	1	1	1	1
12	As very light pressure, palpitation occasionally	R	4	1.89	4	2	2	1	1	1	1	2	3
13	Tingling and a bit like touching sandpaper	L	4.5	3.44	5	4	1	1	4	1	5	5	5
14	Tingling and rubbing as if stimulation	L	3.5	2.67	2	3	1	3	4	1	4	3	3
15	Tingling	R	3.5	1.89	3	4	4	1	1	1	1	1	1
16	Sense of changes in temperature, particularly heat	R	4	1.33	1	1	3	1	1	1	2	1	1
17	Slight tingling sensation	R	4	1.00	1	1	1	1	1	1	1	1	1
18	Prickling and pulses	R	4	1.56	2	1	1	1	1	1	3	3	1
19	Blood pulse in the center of the finger and tingling	L	4	2.00	3	1	1	3	1	1	3	2	3
20	I could evoke the feeling of the sponge, I also felt tingling	L	2.5	1.44	3	3	1	1	1	1	1	1	1
21	Movement and enlargement	R	4.5	1.22	2	2	1	1	1	1	1	1	1
22	Feeling pressure pulsations slightly in the yolks	L	3.5	1.78	3	4	3	1	1	1	1	1	1
23	Temperature fluctuations	R	3.5	1.44	1	2	3	2	1	1	1	1	1
24	I felt like the energy flows into my finger as stimulating untouched, but feeling something very similar	L	5	2.00	3	3	1	4	3	1	1	1	1
25	I could feel pulsations and tingling	R	4	1.56	3	4	1	1	1	1	1	1	1
26	Some tingling in moments of attention, pulsation	L	4.5	1.33	1	4	1	1	1	1	1	1	1
27	Vibrating or pulsing	R	4	2.00	1	1	4	1	4	1	1	4	1
28	I felt it was a little big and sometimes it stung	R	4	1.78	4	1	4	1	1	1	2	1	1
29	Palpitation, heart rate	L	3.5	2.33	3	4	4	1	2	1	3	1	2
30	Throbbing thumb and felt sleepy, numbness strong, feeling hot, stab	R	3.5	2.11	5	5	1	3	1	1	1	1	1
31	Feeling like a wave that came and went and a sensation that the finger felt bigger than normal and a hot feeling in the finger	L	3.5	1.56	2	5	1	1	1	1	1	1	1
32	I felt bigger and hot, it tingled	L	3	1.44	4	1	1	2	1	1	1	1	1
33	Pulsation, tingling and my finger was clear and floated	L	4	2.22	4	3	4	3	1	1	1	1	2
34	Slightly heavier than the other fingers	L	4.5	2.67	5	1	4	4	1	1	4	3	1

The Phenomenology Questionnaire includes a free report of subjective sensations felt during the *Spontaneous Sensation Condition* and a quantitative part Likert scale (1 no sensation to 5 intense sensation) assessment to evaluate the intensity of *Attentional Strength* (A: Attention) and *Specific Sensory Quale experienced*: (P) *Pulsation*, (V) *Vibration*, (E) *Enlargement*, (H) *Heat*, (C) *Cold*, (Sh) *Shrinkage*, (I) *Itching*, (St) *Stinging*, (N) *Numbness*. L = Left,R = Right. The mean for all *quale* experienced by each subject is shown in (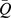). Accordingly (A) and (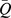) were used to model the respective regressors.

During the scanning an examiner closely monitored the subject's thumb to ensure there was no motion. If there was any perceptible movement the run was discarded. Only six runs from 3 subjects (all right handed) were discarded due to involuntary thumb movement, and the results presented were obtained from the remaining 34 subjects.

### Imaging protocol

fMRI imaging was performed on a 3.0T GE MR750 instrument (General Electric, Waukesha, WI) using a 32-channel head coil. Functional imaging included 35 axial slices, acquired using a T2*-weighted EPI sequence with TR/TE 3000/40 ms, a 64×64 matrix and 4-mm slice thickness, resulting in a 4×4×4 mm^3^ isometric voxel. High-resolution structural 3D-T1-weighted images were acquired for anatomical localization (resolution of 1×1×1 mm^3^, TR = 2.3 sec, TE = 3 ms) covering the whole brain. The images were acquired with an acceleration factor = 2.

### Quantitative evaluation of the Phenomenology Questionnaire

Attention *strength* towards each thumb was assessed with a Likert scale ranging from weak attention (1) to strong attention (5). Subject's subjective sensations scores for attention strength were used as covariates to model the attention regressor. A pilot study performed where volunteers were instructed to focus their attention on either thumb and generate an unrestricted phenomenological description revealed that the most frequently used adjectives were: *pulsation*, *vibration*, *enlargement*, *heat*, *cold*, *shrinkage*, *itching*, *stinging*, and *numbness*. Thus, these were the adjectives used in the subjective sensations Likert scale assessment ranging from no sensation (1) to intense sensation (5). The mean subjective sensation for all these nine somatosensory sensations was used as the covariate to model the *qualia* regressor (see row 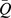 in Table 1 in Results section for details).

### Image processing and statistical analyses

Functional image datasets were processed and analyzed with FSL 4.1.5 (FMRIB's Software Library, www.fmrib.ox.ac.uk/fsl) [27].

#### Preprocessing

The skull and other non-brain areas were extracted from the anatomical and functional scans using the script brain extraction tool (BET) of FSL, motion correction using MCFLIRT [28], spatial smoothing using a Gaussian kernel of FWHM 6 mm, mean-based intensity normalization, and nonlinear highpass temporal filtering. Extracted brains of all participants were linearly registered into the brain-extracted MNI152template using a linear spatial transformation function.

#### First-level fMRI analysis

Statistical analysis was performed with FMRI Expert Analysis Tool using FMRIB's Improved Linear Model (FEAT FILM) Version 5.98 with local autocorrelation correction contrasts with a significance threshold criterion of *Z*>2.3 with a cluster significance threshold of *P* **<**0.05 corrected for multiple comparisons [29] and using the canonical hemodynamic response function (HRF) convolved with a function longer in duration to model the entire blocks and its time derivative as basic functions. The model included the following regressors with their corresponding HRF and their temporal derivatives: *Touch-Stimulus* and *Spontaneous-Sensation* as well as stimulation-aftereffect per thumb, with motion parameters controlled for in the model. The *Touch-Stimulus* regressor was modeled to fit a transient response curve in accord with previous somatosensory habituation reports [30], [31] where somatosensory cortex activation peaked around 6 sec after the onset of the stimulation and then exponentially returned to baseline for the rest of the block. In this manner it was ensured that only the touch-related processes were identified and measured. The *Spontaneous-Sensation* regressor was modeled to fit the last 30 sec of the block, as this would have stronger correspondence to the subjective ratings (see Experimental design), and the first 30 sec were modeled as dummy condition and discarded. Although all four conditions were considered in the GLM, only the response obtained for the *Spontaneous-Sensation Condition* of the last thumb of each participant was assessed and correlated with the subject's *Subjective Sensation* scores obtained from the *Phenomenology Questionnaire*. The rationale is that, although two functional runs were conducted (one for each thumb), the *Phenomenology Questionnaire* was only conducted once at the end of the session. Due to the recency effect, responses to this *Questionnaire* are more applicable to the last thumb stimulated, so only data acquired from the last functional run were analyzed with the *Questionnaire* data.

#### Group-level Subjective-Sensation analysis

To identify activations at the group-level related to attention strength and subjective-sensation for somatosensory experiences, a subjective-sensation analysis using FLAME (FMRIB's Local Analysis of Mixed Effects) was conducted using subject's subjective sensations scores as covariates to model both attention and somatosensory experience regressors (see the Quantitative evaluation of the phenomenology questionnaire and rows *A* and 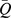 of Table 1). All group Z statistical images were thresholded at Z>2.3 (p<0 .05) to define contiguous voxel clusters. The FSL cluster correction for multiple comparisons (Gaussian-random field theory based) was set at p<0.05, whole brain correction (http://www.fmrib.ox.ac.uk/fsl) [29]. Because we did not find any frontal activation at this threshold as previously hypothesized (see Introduction), we additionally performed an exploratory whole-brain group-level analyses using an uncorrected p-value of p<0.001 with a minimum cluster size threshold (k) of 15 voxels [32], [33]. This statistical threshold is in line with the recommendations for such complex and subtle cognitive processes, as used in previous social and affective neuroscience studies [33]. Subsequently, except where indicated, and due to the documented importance of the frontopolar prefrontal cortex in the integration of multiple separate cognitive processes in the service of higher-order behavioral goals like self referential processes (i.e. mentalizing) and attention [11], we specifically explored this region using a small-volume-correction through a region of interest (ROI) approach. The frontopolar prefrontal ROIs were based on the peak activation of this exploratory whole-brain group-level analyses and the results reported in the meta-analysis in Gilbert et all 2006 that specifically relate to left frontopolar cortex activation either during attention [34]–[37] or during self referential processes (i.e. metalizing) [11]–[13]. The ROIs were defined by merging individually created ROIs of 5 voxel (10 mm) diameter spheres (∼131 mm^3^) around each of the documented peak coordinates and our own results in order to obtain oblong ROI volumes for a) Attention of k = 725 voxels (1450 mm^3^) and b) Subjective Sensation of k = 500 voxels (1000 mm^3^) covering the left frontopolar prefrontal cortex associated with these processes (ROIs were constructed in the 2 mm MNI-152 template). The statistical significance for the ROI analysis were corrected for multiple comparisons using the false discovery rate (FDR) correction as implemented in FSL [38]. The FDR procedure ensures that on average no more than 5% of activated voxels for each contrast are expected to be false positives. The resulting peak voxel activation for either regressor was used to calculate the percent changes of BOLD signal in each subject using Featquery (part of FSL 4.1.5). These signal changes were then correlated with the subject's individual specific subjective attention strength or mean somoatosensory qualia scores of the Lickert scale using Spearman's rank correlation coefficient (see rows *A* and 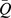 of Table 1). Results were projected onto the surface representation of the MNI-152 template with the Freesurfer suite (http://surfer.nmr.mgh.harvard.edu/) [39] for visualization purposes.

## Results

### Qualitative evaluation of the phenomenology questionnaire

Subject's answers for the *Phenomenology Questionnaire* during the *Spontaneous-Sensation Condition* are shown in Table 1. All subjects experienced and spontaneously expressed their subjective sensations.

### Spontaneous-Sensation analysis

**Figure 2:**
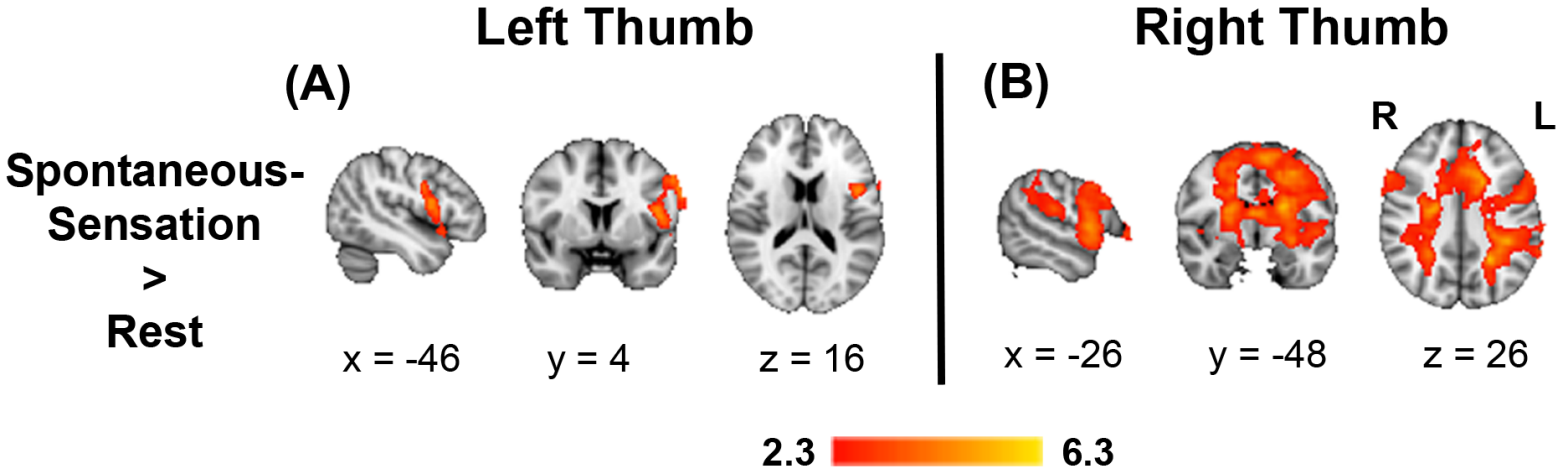
Spontaneous-Sensation analysis: Overall activations associated with focusing of attention during the different phases of the experimental paradigm. A) Focusing attention on Spontaneous-Sensation of the left thumb. B) Focusing of attention on Spontaneous-Sensation of the right thumb. All statistical maps had a significance threshold of Z>2.3, with a cluster significance threshold of p<0.05 (corrected for multiple comparisons). Images are presented in radiological convention and mapped to the MNI-152 template.

Sixty-eight runs (34 right thumb and 34 left thumb) from 34 subjects were included in the analysis. Figure 2 shows that, compared with the resting task-free condition (neither external touch-stimuli nor spontaneous sensations), focusing of attention to *Spontaneous-Sensation* showed a group activation where the peak MNI coordinates for the right thumb (Figure 2B) were found in the left primary somatosensory cortex (BA 3b: X = −58 mm, Y = 6 mm, Z = 14 mm), bilateral secondary somatosensory cortices (SII: 34, 2, 20 and −42, −2, 12), left premotor cortex (BA 6: −2, 6, 52), left parietal lobe (PL: −26, −48, 26), left Broca's area (BA 44: −48, 4, −2), anterior cingulate cortex (BA 32: −18, 14, 28) and right insula (BA 13: 38, 10, 2). Focusing attention on *Spontaneous-Sensation* of the left thumb (Figure 2A), showed activations in the left primary somatosensory cortex (BA 3a: −46, 4, 16), left premotor cortex (BA 6: −56, 10, 42), and left Broca's area (BA 44: −50, 6, 8). Coordinates of peak activation, cluster size and z-values for this and all subsequent contrasts are shown in Table 2.

**Table 2:** Peak voxel activation for all experiments.

Contrast	Anatomical Location	MNI Peak coordinates (mm) x, y, z	Z-score
**Spontaneous-Sensation**			
***Right Thumb***			
SS>rest^a^	Ba3b L, SI L	−58, 6, 14	3.23
	SII R	34, 2, 20	3.29
	SII L	−42, −2, 12	5.24
	Ba6 L, PMC	−2, 6, 52	3.82
	PL L	−26, −48, 26	5.28
	Ba44 L, Broca	−48, 4, −2	3.81
	Ba32, ACC	−18, 14, 28	4.62
	Ba13 R, Insula	38, 10, 2	5.16
***Left Thumb***			
SS > rest^a^	Ba3b L, SI L	−46, 4, 16	3.39
	Ba6 L, PMC	−56, 10, 42	4.10
	Ba44 L, Broca	−50, 6, 8	3.38
**Subjective-Sensations**			
Attention^b^	Ba 10 L, fpPFC^b^	−4, 66, −4	3.78
Subjective Somatosensory experiences^a^^,^ b			
	Ba 10 L, fpPFC^b^	−20, 72, 8	3.16
	Ba 2 R, SI^a^	28, −42, 62	2,83
	Ba 6 R, PMC	50, 0, 28	3.47
	Ba 7, R, Precun	8, −64, 52	3.39
	Ba 38 L, TPC^a^	−32, 2, −18	3.49
	BA39 R, IPL^a^	46, −70, 32	3.93
	Hippo R^a^	30, −26, −14	3.94
	Insula R^a^	38, −8, 4	3.73
	Amygdala R^a^	26, −8, −18	4.98

Peak activations for Spontaneous- and Subjective-sensations analysis conditions. SS: *Spontaneous-Sensation*, R: right, L: left, Ba: Brodmann area, ACC: anterior cingulate cortex, SI: primary somatosensory cortex, SII: secondary somatosensory, fpPFC: frontopolar prefrontal cortex, MFG: medial frontal gyrus, SFG: superior frontal gyrus, Hippo: hippocampus, TPC: temporopolar cortex, Precun: precuneus, IPL:inferior parietal lobe.

a = cluster corrected with threshold z>2.3, p<0.05;

b = small-volume-correction using False Discovery Rate (FDR) with a p<0.05 as implemented in FSL [38].

The activations found during the *Touch-Stimulus Condition* and their relation to the activations during the *Spontaneous-Sensation Condition* are detailed in a separate communication [21]. It is relevant to mention here that the contralateral activation of the somatosensory cortex (BA 3a/b corresponding to the hand area) obtained during the *Touch-Stimulus Condition* was also observed during the *Spontaneous-Sensation Condition*. Additionally, a left parieto-frontal activation was detected in the first-level analysis in the right-handed subjects during the *Spontaneous-Sensation Condition*. This prompted us to include a sample of 14 left-handed individuals for a statistically suitable comparison, but no differences between right and left-handed subjects were found after analyzing right and left thumbs separately and between groups for details please refer to [21]. Thus, we considered both hand-dominance groups as statistically similar and the left parieto-frontal activation as a result of top-down attentional mechanisms for a discussion on this please see [21].

### Subjective-Sensation analysis

#### Attention

**Figure 3:**
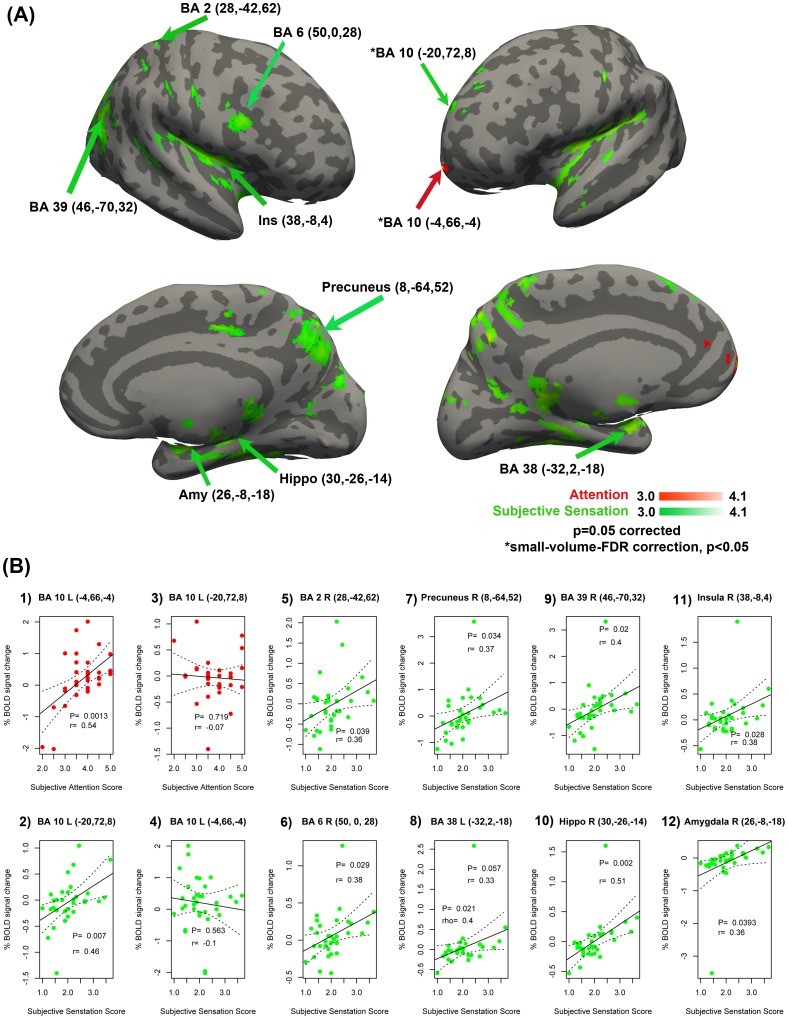
Subjective-Sensation analysis: Significant activations and correlations for the covariates from the *Phenomenology Questionnaire.* A) *Attention* as a covariate revealed left ventral frontopolar prefrontal cortex (BA10 in red); *Subjective somatosensory experience* mean as a covariate revealed (in green) left dorsal frontopolar prefrontal cortex (BA10 in green), right primary somatosensory cortex (BA2), right premotor cortex (BA 6), precuneus (BA 7), left temporopolar cortex (BA 38), right inferior parietal lober (BA 39), right hippocampus, right insula and right amygdala, and. B) Spearman's rank correlations of subjective sensation scores with % BOLD signal change of peak voxels for 1) Subjective Attention score vs. ventral BA10 L, 2) Subjective Somatosensory Experiences vs. ventral BA 10 L, 3) Subjective Attention score vs. dorsal BA10 L, 4) Subjective Somatosensory Experiences vs. dorsal BA 10 L, 5) Subjective Somatosensory Experiences vs. BA 2 R, 6) Subjective Somatosensory Experiences vs. BA 6 L, 7) Subjective Somatosensory Experiences vs.BA 7 R, 8) 6) Subjective Somatosensory Experiences vs. BA 38 L, 9) Subjective Somatosensory Experiences vs. BA 39 R, 10) Subjective Somatosensory Experiences vs. Hippocampus R, 11) Subjective Somatosensory Experiences vs. Insula R, 12) Subjective Somatosensory Experiences vs. Amygdala R. Coordinates shown are X, Y, Z in mm for the MNI152 template. Activations have a significance threshold of Z**>**2.3, with a cluster significance threshold of p**<**0.05 (corrected for multiple comparisons) except for *BA10 = small-volume-FDR-correction with p<0.05. All correlations were assessed with the Pearson product-moment correlation and assessed for outliers using Spearman's rank-order correlation. Dashed lines indicate 95% confidence intervals.

left frontopolar prefrontal cortex (ventral portion) (BA 10: −4, 66, −4; Z = 3.78, p<0.05, small-volume-FDR-corrected; red cluster in Figure 3A) was active for attention as covariate and the percentage BOLD signal change correlated positively with the subjects' subjective attention strength reports (r(32) = 0.54, p = 0.0013, Figure 3B.1) but not for subjects' subjective somatosensory experience reports (r(32) = −0.1, p = 0.563, Figure 3B.4).

#### Subjective Somatosensory Experiences

Activity in several regions covaried with subjective somatosensory experiences (Figure 3A, green clusters, all clusters corrected p<0.05). These regions include the left frontopolar prefrontal cortex (dorsal portion) (BA10: −20, 72, 8), right primary somatosensory cortex (BA 2: 28, −42), right premotor cortex (BA 6: 50, 0, 28), right precuneus (BA 7: 8, −64, 52), left temporopolar cortex (BA 38: −32, 2, −18), right inferior parietal lobe (BA 39: 46, −70, 32), right hippocampus (30, −26, −14), right insula (38, −8, 4), and right amygdala (26, −8, −18). Additionally, the percentage BOLD signal change correlated positively with the subjects' subjective somatosensory experience reports (i.e. BA10: r(32) = 0.46, p = 0.007, Figure 3B.2; BA 2: r(32) = 0.36, p = 0.039, Figure 3B.5; BA 6: r(32) = 0.38, p = 0.029, Figure 3B.6; Precuneus: r(32) = 0.37, p = 0.034, Figure 3B.7; BA 38: r(32) = 0.33, p = 0.57, ρ = 0.4, p = 0.21, Figure 3B.8; BA 39: r(32) = 0.4, p = 0.02, Figure 3B.9; Hippocampus: r(32) = 0.51, p = 0.002, Figure 3B.10; Insula: r(32) = 0.38, p = 0.028, Figure 3B.11; Amygdala: r(32) = 0.36, p = 0.039, Figure 3B.12). Additionally, ventral BA 10 (−20, 72, 8) did not correlate with subjects' subjective attention strength reports (r = −0.07, p = 0.719, Figure 3B.3). To check for outliers we ran a non-parametric correlation test for all the brain areas, i.e. Spearman's rank-order correlation, which only showed a significant change for BA 38 (see above and Figure 3B.8).

## Discussion

After verifying in 34 healthy volunteers that sustained attention directed to the spontaneous sensations of either thumb in the absence of any external stimuli effectively activates brain somatosensory areas, the present results show that corresponding subjective somatosensory experiences correlate with left dorsal frontopolar prefrontal cortex, right primary somatosensory cortex, left temporopolar cortex, right inferior parietal lobe, right hippocampus, right insula and right amygdala activations. Therefore, the main hypothesis of this work was largely corroborated with the additional finding that the left frontopolar prefrontal cortex (BA 10) and the temporopolar cortex (BA 38), in conjunction with primary somatosensory (BA 2), cortex, premotor cortex (BA 6), precuneus (BA 7), inferior parietal lobe (BA 39), hippocampus, insula and amygdala are involved in general spontaneous subjective somatosensory experiences.

The results show that the frontopolar prefrontal cortex has functional subdivisions, updating previous theories [11]. In particular, we show that the dorsal part of the frontopolar prefrontal cortex is involved during subjective sensory experiences known as *qualia* [6]–[8] and that it is coupled with other brain areas during this process. Hence, contributing to narrow down the individual brain structures involved [9], [10], [40]. In particular, our results agree with Feinstein et al. [6] in terms that the magnitude and time course of activation within the frontopolar prefrontal cortex, medial prefrontal cortex, and the anterior cingulate predict whether information is consciously perceived or slips away unnoticed. Other studies also report signal increases in frontopolar prefrontal cortex during different self-referential processing tasks [11]–[13]. It has also been shown that synchronic frontal gamma patterns (around 40 Hz) emerge with the recognition of a 3D object from an auto-stereogram and this pattern occurred only when subjects were readily expecting the arrival of the concealed visual object (26).

We also found that the left temporopolar cortex (BA38), together with the frontopolar cortex, becomes active during both attention mechanisms and subjective experience. Since the temporopolar cortex is a convergence zone where information from sensory, association, and limbic systems is integrated [41], [42]; this activation may relate to the awareness and conscious processing of the affective component of somatosensory experiences. In agreement with this interpretation, Ramsøy et al. [14] found that object encoding evokes bilateral activations of temporopolar, perirhinal, parahippocampal cortices, hippocampus and amygdala, while D'Argembeau et al. [43] found that the temporopolar cortex along with dorsomedial prefrontal cortex, left anterior middle temporal gyrus, and right cerebellum is implicated in reflective tasks pertaining to self, another person, and social issues.

Besides frontopolar and temporopolar activation, in the present study other areas appeared to be involved in the retrieval and processing of somatosensory experiences, i.e., primary somatosensory cortex, premotor cortex, precuneus, inferior parietal lobe, hippocampus, insula and amygdala. The combined activity of these areas probably supports conscious perceptual and phenomenological awareness [44], [45]. Consequently, pimary somatosensory cortex activation suggests its causal involvement due to the nature of the attended somatosensory experiences [21], [45]. Parietal and premotor cortices have been implicated in multisensory integration, embodiment, localization and self-attribution of body parts [46]–[49] and insula activation has been implicated in the integration of interoceptive and exteroceptive signals to construct the mental self [49], [50] and amygdala activation has been found coupled to frontal brain regions when subjects involve in self-related processing [43] and is probably a key node involved in self-referential emotion processing [51]–[53]. Finally, autobiographical memory and past experiences relate to consciousness of one self, which requires hippocampal processing [54]–[57]. Even though the instructions in our study focus on actual somatosensory experiences, the activations detected in these brain areas suggests an underlying neurocognitive requirement of body-ownership and self-consciousness. Finally, the noteworthy finding that primary somatosensory cortex is activated in the absence of external stimulation by the focusing of attention on spontaneous sensory qualia verifies that selective attention controlled by top-down cognitive processes enhance bottom-up qualitative processes of somatosensory/external and proprioceptive/internal nature that normally do not elicit primary somatosensory cortex activity in absence of stimuli [17]–[20]. This spontaneously-elicited somatosensory activity is accompanied by phenomenological somatosensory qualitative experiences or *qualia*, some of the most characteristic and enigmatic subjective phenomena [58], but suitable to be correlated with objective measures of brain activity [59].

Within a broader perspective, the study of sensory *qualia* intending to match third-person fMRI brain imaging with standardized first-person somatosensory reports constitutes a particular neurophenomenological endeavor to study the neural correlates of qualitative subjective experience. In the light of the present results, the precise mechanism for the production or correspondence of subjective sensory experiences in the detected neural networks remains a challenging, but perhaps a more delimited research question.

## Supporting Information

Data S1 (DOCX)

## References

[1] Thompson E (2004) Life and mind: From autopoiesis to neurophenomenology. A tribute to Francisco Varela. Phenomenol Cogn Sci 3: 381–398 10.1023/B:PHEN.0000048936.73339.dd

[2] Varela FJ (1996) Neurophenomenology: A methodological remedy for the hard problem. Journal of Consciousness Studies 3 (4) 330–349.

[3] Díaz JL (2007) La conciencia viviente. Fondo de Cultura Económica.

[4] Díaz JL (2013) A Narrative Method for Consciousness Research. Front Hum Neurosci 7. 10.3389/fnhum.2013.00739 PMC3821450 24265610

[5] Nagel T (1974) What is it like to be a bat? Philos Rev 435–450.

[6] Feinstein JS, Stein MB, Castillo GN, Paulus MP (2004) From sensory processes to conscious perception. Conscious Cogn 13: 323–335 10.1016/j.concog.2003.10.004 15134763

[7] Northoff G (2003) Qualia and the Ventral Prefrontal Cortical Function Neurophenomenological Hypothesis. J Conscious Stud 10: 14–48.

[8] Lutz A (2002) Toward a neurophenomenology as an account of generative passages: A first empirical case study. Phenomenol Cogn Sci 1: 133–167.

[9] Damasio AR (1999) *The feeling of what happens: Body and emotion in the making of consciousness*. Mariner Books.

[10] Paradiso S, Johnson DL, Andreasen NC, O'Leary DS, Watkins GL, et al (1999) Cerebral blood flow changes associated with attribution of emotional valence to pleasant, unpleasant, and neutral visual stimuli in a PET study of normal subjects. Am J Psychiatry 156: 1618–1629. 10518175 10.1176/ajp.156.10.1618

[11] Gilbert SJ, Spengler S, Simons JS, Steele JD, Lawrie SM, et al (2006) Functional specialization within rostral prefrontal cortex (area 10): a meta-analysis. J Cogn Neurosci 18: 932–948. 16839301 10.1162/jocn.2006.18.6.932

[12] McCaig RG, Dixon M, Keramatian K, Liu I, Christoff K (2011) Improved modulation of rostrolateral prefrontal cortex using real-time fMRI training and meta-cognitive awareness. Neuroimage 55: 1298–1305. 21147230 10.1016/j.neuroimage.2010.12.016

[13] Brass M, Derrfuss J, von Cramon DY (2005) The inhibition of imitative and overlearned responses: a functional double dissociation. Neuropsychologia 43: 89–98 10.1016/j.neuropsychologia.2004.06.018 15488909

[14] Ramsøy TZ, Liptrot MG, Skimminge A, Lund TE, Sidaros K, et al (2009) Regional activation of the human medial temporal lobe during intentional encoding of objects and positions. Neuroimage 47: 1863–1872. 19362156 10.1016/j.neuroimage.2009.03.082

[15] Asari T, Konishi S, Jimura K, Chikazoe J, Nakamura N, Miyashita Y (2008) Right temporopolar activation associated with unique perception. NeuroImage 41 (1) 145–152 10.1016/j.neuroimage.2008.01.059. 18374603

[16] Christoff K, Ream JM, Gabrieli JDE (2004) Neural Basis of Spontaneous thought Processes. Cortex 40: 623–630 10.1016/S0010-9452(08)70158-8 15505972

[17] Treisman AM, Gelade G (1980) A feature-integration theory of attention. Cognitive Psychology 12 (1) 97–136. 7351125 10.1016/0010-0285(80)90005-5

[18] Sarter M, Givens B, Bruno JP (2001) The cognitive neuroscience of sustained attention: where top-down meets bottom-up. Brain Res Rev 35: 146–160. 11336780 10.1016/s0165-0173(01)00044-3

[19] Buschman TJ, Miller EK (2007) Top-down versus bottom-up control of attention in the prefrontal and posterior parietal cortices. Science 315: 1860. 17395832 10.1126/science.1138071

[20] Petersen SE, Posner MI (2012) The Attention System of the Human Brain: 20 Years After. Annual Review of Neuroscience 35: 73–89 10.1146/annurev-neuro-062111-150525. PMC3413263 22524787

[21] Bauer CC, Díaz JL, Concha L, Barrios FA (2014) Sustained attention to spontaneous thumb sensations activates brain somatosensory and other proprioceptive areas. Brain Cogn 87: 86–96. 24727703 10.1016/j.bandc.2014.03.009

[22] Cahn B, Delorme A, Polich J (2010) Occipital gamma activation during Vipassana meditation. Cogn Process 11: 39–56 10.1007/s10339-009-0352-1 20013298 PMC2812711

[23] Romo R, Brody CD, Hernández A, Lemus L (1999) Neuronal correlates of parametric working memory in the prefrontal cortex. Nature 399: 470–473 10.1038/20939 10365959

[24] Farb NAS, Segal ZV, Anderson AK (2013) Attentional Modulation of Primary Interoceptive and Exteroceptive Cortices. Cereb Cortex 23: 114–126 10.1093/cercor/bhr385 22267308 PMC3513954

[25] Oldfield RC (1971) The assessment and analysis of handedness: the Edinburgh inventory. Neuropsychologia 9: 97–113. 5146491 10.1016/0028-3932(71)90067-4

[26] González-Santos L, Mercadillo RE, Graff A, Barrios FA (2007) Del Listado de Síntomas 90 (SCL 90) y Del Inventario de Temperamento y Carácter (ITC). Salud Mental 30: 31.

[27] Smith SM, Jenkinson M, Woolrich MW, Beckmann CF, Behrens TEJ, et al (2004) Advances in functional and structural MR image analysis and implementation as FSL. Neuroimage 23: S208–S219. 15501092 10.1016/j.neuroimage.2004.07.051

[28] Jenkinson M, Smith S (2001) A global optimisation method for robust affine registration of brain images. Medical Image Analysis 5 (2) 143–156. 11516708 10.1016/s1361-8415(01)00036-6

[29] Worsley KJ (2001) Statistical analysis of activation images. Funct MRI Introd Methods 14: 251–270.

[30] Klingner CM, Nenadic I, Hasler C, Brodoehl S, Witte OW (2011) Habituation within the somatosensory processing hierarchy. Behav Brain Res 225: 432–436 10.1016/j.bbr.2011.07.053 21840344

[31] Moulton EA, Keaser ML, Gullapalli RP, Greenspan JD (2005) Regional intensive and temporal patterns of functional MRI activation distinguishing noxious and innocuous contact heat. Journal of Neurophysiology 93 (4) 2183. 15601733 10.1152/jn.01025.2004

[32] Forman SD, Cohen JD, Fitzgerald M, Eddy WF, Mintun MA, et al (1995) Improved assessment of significant activation in functional magnetic resonance imaging (fMRI): use of a cluster-size threshold. Magn Reson Med 33: 636–647. 7596267 10.1002/mrm.1910330508

[33] Lieberman MD, Cunningham WA (2009) Type I and Type II error concerns in fMRI research: re-balancing the scale. Soc Cogn Affect Neurosci 4: 423–428 10.1093/scan/nsp052 20035017 PMC2799956

[34] Fan J, McCandliss BD, Sommer T, Raz A, Posner MI (2002) Testing the efficiency and independence of attentional networks. J Cogn Neurosci 14: 340–347. 11970796 10.1162/089892902317361886

[35] Lepsien J, Pollmann S (2002) Covert reorienting and inhibition of return: an event-related fMRI study. J Cogn Neurosci 14: 127–144. 11970781 10.1162/089892902317236795

[36] Pollmann S, Weidner R, Müller HJ, Yves von Cramon D (2006) Neural correlates of visual dimension weighting. Vis Cogn 14: 877–897 10.1080/13506280500196142

[37] Small DM, Gitelman DR, Gregory MD, Nobre AC, Parrish TB, et al (2003) The posterior cingulate and medial prefrontal cortex mediate the anticipatory allocation of spatial attention. Neuroimage 18: 633–641. 12667840 10.1016/s1053-8119(02)00012-5

[38] Genovese CR, Lazar NA, Nichols T (2002) Thresholding of statistical maps in functional neuroimaging using the false discovery rate. Neuroimage 15: 870–878. 11906227 10.1006/nimg.2001.1037

[39] Dale AM, Fischl B, Sereno MI (1999) Cortical surface-based analysis: I. Segmentation and surface reconstruction. Neuroimage 9: 179–194. 9931268 10.1006/nimg.1998.0395

[40] Lutz A, Lachaux JP, Martinerie J, Varela FJ (2002) Guiding the study of brain dynamics by using first-person data: Synchrony patterns correlate with ongoing conscious states during a simple visual task. Proc Natl Acad Sci U S A 99: 1586–1591. 11805299 10.1073/pnas.032658199 PMC122234

[41] Pascual B, Masdeu JC, Hollenbeck M, Makris N, Insausti R, et al (2013) Large-Scale Brain Networks of the Human Left Temporal Pole: A Functional Connectivity MRI Study. Cereb Cortex bht260 10.1093/cercor/bht260 PMC4318532 24068551

[42] Morán MA, Mufson EJ, Mesulam M-M (1987) Neural inputs into the temporopolar cortex of the rhesus monkey. J Comp Neurol 256: 88–103 10.1002/cne.902560108 3819040

[43] D'Argembeau A, Collette F, Van der Linden M, Laureys S, Del Fiore G, et al (2005) Self-referential reflective activity and its relationship with rest: a PET study. Neuroimage 25: 616–624. 15784441 10.1016/j.neuroimage.2004.11.048

[44] Sadaghiani S, Kleinschmidt A (2013) Functional interactions between intrinsic brain activity and behavior. NeuroImage doi:http://www.sciencedirect.com/science/article/pii/S105381191300459X 10.1016/j.neuroimage.2013.04.100 23643921

[45] Rees G (2013) Neural correlates of consciousness. Ann N Y Acad Sci 1296: 4–10 10.1111/nyas.12257 23991638 PMC4166676

[46] Guterstam A, Gentile G, Ehrsson HH (2013) The Invisible Hand Illusion: Multisensory Integration Leads to the Embodiment of a Discrete Volume of Empty Space. J Cogn Neurosci 25: 1078–1099 10.1162/jocna00393 23574539

[47] Brozzoli C, Gentile G, Ehrsson HH (2012) That's Near My Hand! Parietal and Premotor Coding of Hand-Centered Space Contributes to Localization and Self-Attribution of the Hand. J Neurosci 32: 14573–14582 10.1523/JNEUROSCI.2660-12.2012 23077043 PMC6621451

[48] Freton M, Lemogne C, Bergouignan L, Delaveau P, Lehéricy S, et al (2014) The eye of the self: precuneus volume and visual perspective during autobiographical memory retrieval. Brain Struct Funct 219: 959–968 10.1007/s00429-013-0546-2 23553546

[49] Cabanis M, Pyka M, Mehl S, Müller BW, Loos-Jankowiak S, et al (2013) The precuneus and the insula in self-attributional processes. Cogn Affect Behav Neurosci 13: 330–345 10.3758/s13415-012-0143-5 23297009

[50] Seth AK (2013) Interoceptive inference, emotion, and the embodied self. Trends Cogn Sci 17: 565–573 10.1016/j.tics.2013.09.007 24126130

[51] Northoff G, Heinzel A, de Greck M, Bermpohl F, Dobrowolny H, et al (2006) Self-referential processing in our brain? A meta-analysis of imaging studies on the self. Neuroimage 31: 440–457. 16466680 10.1016/j.neuroimage.2005.12.002

[52] Frewen PA, Lundberg E, Brimson-Théberge M, Théberge J (2013) Neuroimaging self-esteem: a fMRI study of individual differences in women. Soc Cogn Affect Neurosci 8: 546–555 10.1093/scan/nss032 22403154 PMC3682439

[53] Schwarz KA, Wieser MJ, Gerdes ABM, Mühlberger A, Pauli P (2013) Why are you looking like that? How the context influences evaluation and processing of human faces. Soc Cogn Affect Neurosci 8: 438–445 10.1093/scan/nss013 22287265 PMC3624952

[54] Smith C, Squire LR (2005) Declarative Memory, Awareness, and Transitive Inference. J Neurosci 25: 10138–10146 10.1523/JNEUROSCI.2731-05.2005 16267221 PMC1457087

[55] Prebble SC, Addis DR, Tippett LJ (2013) Autobiographical memory and sense of self. Psychol Bull 139: 815–840 10.1037/a0030146 23025923

[56] Martinelli P, Sperduti M, Piolino P (2013) Neural substrates of the self-memory system: New insights from a meta-analysis. Hum Brain Mapp 34: 1515–1529 10.1002/hbm.22008 22359397 PMC6870171

[57] Behrendt R-P (2013) Hippocampus and consciousness. Rev Neurosci 24: 239–266 10.1515/revneuro-2012-0088 23585178

[58] Campana F, Tallon-Baudry C (2013) Anchoring visual subjective experience in a neural model: The coarse vividness hypothesis. Neuropsychologia 51: 1050–1060 10.1016/j.neuropsychologia.2013.02.021 23499720

[59] Garrison KA, Scheinost D, Worhunsky PD, Elwafi HM, Thornhill IV TA, et al (2013) Real-time fMRI links subjective experience with brain activity during focused attention. NeuroImage 81: 110–118 10.1016/j.neuroimage.2013.05.030 23684866 PMC3729617

