# Supplementary material for: Subjective Somatosensory Experiences Disclosed by Focused Attention: Cortical-Hippocampal-Insular and Amygdala Contributions
Source: PLoS One. 2014 Aug 28;9(8):e104721. doi: 10.1371/journal.pone.0104721 (PMC4148258; doi:10.1371/journal.pone.0104721)
Supplement: Data S1 — (DOCX) [file pone.0104721.s001.docx]

All the MR data used in this research is available on request. To request access to our public server and instructions for downloading the data please contact:

Fernando A. Barrios, Ph.D.

[fbarrios@unam.mx](mailto:fbarrios@unam.mx)

Luis Concha, Ph.D.

[lconcha@unam.mx](mailto:lconcha@unam.mx)

or

Leopoldo González-Santos

[lgs@unam.mx](mailto:lgs@unam.mx)

+52(442)238-1053

+52(442)238-1054

FAX +52(442)238-1046

Instituto de Neurobiología

Universidad Nacional Autónoma de México

Campus Juriquilla

Blvrd Juriquilla 3001

Querétaro, QRO

76230

México
